# Coprological and Molecular Analyses of Ruminant Farms in Québec, Canada, Show a Variable Efficacy of Ivermectin Against Gastro-Intestinal Nematodes

**DOI:** 10.3390/pathogens14100984

**Published:** 2025-09-28

**Authors:** Behrouz Rezanezhad-Dizaji, Levon Abrahamyan, Marjolaine Rousseau, Pablo Godoy

**Affiliations:** Faculty of Veterinary Medicine, University of Montreal, Saint-Hyacinthe, QC J2S 2M2, Canada; behrouz.rezanezhad.dizaji@umontreal.ca (B.R.-D.); levon.abrahamyan@umontreal.ca (L.A.); marjolaine.rousseau@umontreal.ca (M.R.)

**Keywords:** gastro-intestinal nematodes, anthelmintic resistance, ivermectin, grazing ruminants, drug efficacy

## Abstract

Gastro-intestinal nematodes (GINs) are still of great concern in grazing ruminants, such as camelids, ovines and caprines, affecting animal health and productivity. This is mainly due to the development of anthelmintic resistance (AR) to the compounds used long term, without much evaluation on their efficacy, including ivermectin (IVM), the most used anthelmintic drug in livestock. The aims of this study were to determine the efficacy of IVM and identify which GIN species are affecting different ruminant farms in Quebec (QC), Canada. Firstly, we collected fecal samples from six farms with different ruminant species (camelids, goats and sheep) before and after IVM treatment when applicable, analyzing them by Fecal Egg Count (FEC) and further assessments on IVM efficacy through the Fecal Egg Count Reduction Test (FECRT). In addition, molecular analyses were conducted using PCR, targeting the ITS-2 and COX-1 genes to identify GIN species. FECRT was applied only for three farms, showing that variable results with optimal efficacy (ranging from 95.5–100%) were obtained in only one farm, whereas on the other two farms, FECRT exhibited reduced efficacy, suggesting the development of IVM resistance. Among the GIN species found, *Haemonchus contortus* and *Trichostrongylus vitrinus* were identified in most of the farms, being present in sheep, goat, llama and alpaca farms, whereas *Teladorsagia circumcincta* was identified only in sheep and llama samples from four farms but not in alpaca samples. *Trichostrongylus axei* and *Chabertia ovina* were present in two farms (sheep and sheep and llamas). *Oesophagostomum venulosum* was detected in one sheep and one alpaca farm. Only one sheep farm was positive for *Trichostrongylus colubriformis* and *Cooperia curticei*. Also, *Nematodirus* spp. and *Trichuris* spp. were found in four farms, including sheep and camelids. In addition, three other species were found in camelids, including *Camelostrongylus mentulatus* (only in the llama samples), whereas *Lamanema chavezi* and *Marshallagia marshalli* were identified in one alpaca farm. Therefore, our work reports evidence of an uneven efficacy of IVM against GINs from ruminant farms, including the most likely emergence of IVM resistance. The diversity of GIN species found in ruminant farms in QC along with the inconsistent IVM efficacy are helpful information for veterinarians and animal producers in setting an optimal parasite management programs, including the proper use of IVM and alternative anthelmintic drugs to control these pathogens in grazing livestock.

## 1. Introduction

Veterinary nematodes affect grazing ruminants all around the world [1,2]. The effect of their parasitism has significantly impacted the small ruminants’ industry in several countries [3,4,5]. In North America, epidemiological studies have identified the presence of multiple species of parasitic nematodes, including the strongylid suborder in grazing livestock and ruminant wildlife [6,7,8,9]. Conventionally, the control of parasitic nematodes in agricultural systems has relied on the use of anthelmintic formulations, a practice that has contributed to the emergence of anthelmintic resistance (AR) in gastro-intestinal nematodes (GINs) [10]. In Canada, the main classes of anthelmintic drugs used to control GINs in livestock include macrocyclic lactones (MLs) and benzimidazoles (BZs) [11]. MLs are broad-spectrum anthelmintics that act by binding irreversibly to nematode Glutamate-gated chloride channels (GluCls), leading to a flaccid paralysis and further death [10], whereas BZs bind to the nematode’s β-tubulin, inducing its depolymerization and cytoskeleton disruption [10].

The diagnosis of GINs as well the assessment of drug efficacy and AR in farmed ruminants are typically performed through the fecal egg count (FEC) technique [12]. The development of new technologies has enhanced the identification of GIN species from using microscopic observation of eggs and larval morphology [13] to molecular techniques, such as PCR and deep-amplicon sequencing (DNA bar cording) [14,15]. However, besides the fecal egg count reduction test (FECRT) and assays for the SNP associated with BZ resistance, there are no standardized alternative methods or reliable markers to measure AR in GINs for other anthelmintic classes [16].

*Haemonchus contortus* is among the GIN species that parasitize many grazing ruminants [17]. This harmful blood-feeding helminth is the most successful species of parasitic nematode in developing AR to both old and new anthelmintic formulations [18,19,20]. *H. contortus* has been previously described in some parts of North America, where it affects mainly small ruminants and New World camelids, such as alpacas and llamas, and occasionally beef cattle [21,22,23]. In some provinces of Canada, research reports, mainly from sheep and goat farms, indicate the prevalence of GIN species, including *H. contortus* isolates, with evidence of AR to BZs [24,25]. Resistance to MLs, most commonly, resistance to ivermectin (IVM), has been identified in GINs from beef cattle in Western Canada [26].

Based on the widespread use of IVM to control parasitic nematodes in livestock raised on pastures, in the present study, we aimed to identify the GIN species that affect different grazing ruminants in Québec (Eastern Canada) and to assess the efficacy of IVM as the most common anthelmintic drug used to control parasitic nematodes.

## 2. Materials and Methods

### 2.1. Ruminant Farms and Sample Collection for IVM Efficacy Assessment

In total, 6 grazing ruminant farms were included in our study (Table 1) as follows: farm 1 contained 11 sheep of 2 years old (Y.O.), but only 9 were treated with IVM. Farm 2 corresponds to 9 sheep that were 2 Y.O., all treated with IVM. Farm 3 included 6 goats (5 adults that were 2 Y.O. and 1 kid) in addition to 2 sheep that were 2 Y.O. No animal was treated with IVM. Farm 4 has two age groups of alpacas: one group of 8 animals aged 1 Y.O. and another group of 7 animals of 2 Y.O. Both groups were treated with a monthly dose of IVM. Farm 5 had a total of 11 alpacas, divided in 9 adults that were 2 Y.O., 1 young adult being 1 Y.O. and 1 cria (6 months old), and no animal received IVM treatment. Farm 6 included 13 animals with no IVM treatment, consisting of 12 adults that were 2 Y.O. and 1 young adult aged 1 Y.O. Farms included in our study came from 3 Québec regions (Centre-Sud, Estrie and Montéregie), running the study between April 2021 and September 2022. Individual fecal samples were collected from the animal’s rectum, whereas pooled samples were collected by veterinarians, joining individual fresh samples from several animals. All fecal samples were placed on sealed and labeled plastic bags and were sent on ice on the same day or the next one (kept refrigerated at 4 °C) to the diagnostic service at FVM. Fecal samples were stored at 4 °C for 24–48 h until coprological analysis. Animals treated in our study received an oral IVM formulation (Ivomec^®^, Boehringer Ingelheim, Burlington, ON, Canada), based on individual weight and ruminant species dosage. Based on initial coprological results indicating a low parasite burden, farm 3 (goats and sheep) and alpaca farms 5 and 6 were not treated with IVM, based on veterinarians’ decision. Following label instructions, sheep received 0.2 mg/kg of IVM. For camelid (alpaca or llama) farms, IVM was administrated as an extra-label oral drench formulation (Ivomec^®^), administering 0.4 mg/kg to domestic camelid species [27]. On small ruminant farms, fecal samples were collected before and 14 days after IVM treatment individually or in pools when applicable, whereas for camelid farms, fecal samples were collected individually from each animal treated or not with IVM. Among the camelid farms, we included alpaca farm 4 that has been using IVM as a monthly preventative treatment against the meningeal worm *Parelaphostrongylus tenuis*, a parasitic nematode of wild ruminants in North America that could severely affect domesticated ruminants [28].

### 2.2. Coprological Analyses

Fecal samples from sheep (farms 1 and 2) were processed following the Wisconsin method, whereas the fecal samples from alpacas were analyzed through the Wisconsin (farms 4, 5 and 6) and Mini-FLOTAC method (only for farms 3 and 4 when the Mini-FLOTAC kit was available), determining FEC and further IVM efficacy [12]. Briefly, from each sample collected from small ruminant farms, three replicates of 3 g of feces were weighed and homogenized in 20 mL of water in a small plastic beaker. Then, each sample replicate was stirred in a 50 mL Falcon tube with the addition of water up to 45 mL. The prepared mix was centrifuged at 850× *g* for 5 min, and the supernatant was discarded. The remaining pellet was resuspended in 5 mL of a saturated sugar solution (specific gravity: 1.30) and transferred to a 15 mL Falcon tube, followed by centrifugation at 350× *g* for 2 min. Finally, a saturated sugar solution was added to the tubes until the formation of a positive meniscus at the top of the tube, and a coverslip was fixed on the tubes [29]. After one h of incubation at RT, the coverslip was picked up and placed on the glass slide for FEC under a compound microscope at 10× magnification. The total number of counted eggs in each slide was divided into three (the weight of feces) to achieve each replicate’s egg per gram (EPG). The mean EPG of three replicates represented the EPG for each sample pre- or post-IVM treatment when applicable.

Fecal samples from sheep and goats from farm 3 and alpacas from farm 4 were subjected to the Mini-FLOTAC method for FEC [12]. A total of 2.5 g of feces from each sample were weighed and placed in the conical collector of the fill-FLOTAC. Then, 47.5 mL of a saturated salt solution (specific gravity: 1.2) was added to the fill-FLOTAC container, and the container was screwed closed [29]. The sample was homogenized by gently pumping and circulating the homogenizer pole of the fill-FLOTAC. After, the homogenized sample was transferred to the two chambers of the Mini-FLOTAC disk, avoiding bubbles. Subsequently, the sample was incubated for 10 min, followed by a gentle turn clockwise (90°) of the Mini-FLOTAC disk and further examined under the microscope to count the GIN eggs. The total FEC in two chambers was multiplied by 10 (multiplication factor) to calculate the mean EPG of three replicates per sample.

Hinging on microscopical analyses, GIN eggs were classified into a strongylid-like form and distinct nematode eggs from the genera *Nematodirus* and *Trichuris* [30]. Only FEC from strongylid-like eggs were used to evaluate IVM efficacy [31].

### 2.3. IVM Efficacy Assessment

Based on the initial FEC results from the pre-treatment coprological analyses, only 2 sheep farms (farm 1 and farm 2) that were treated with IVM and only one alpaca farm (farm 4, monthly IVM treatment) were included in the efficacy analysis through FECRT. Given the variation in animal numbers among the ruminant farms subjected to IVM treatment and the paired samples (individual pre- and post-treatment for sheep farm 1 (N = 9) and alpaca farm 4 with 2 subgroups (N = 8 and N = 7)) or the pooled fecal samples at pre-and post-IVM treatment from sheep farm 2 (N = 9), we applied the “clinical protocol” criteria to analyze and interpret the FECRT results on IVM efficacy [31]. As such, we calculated EPG means with a 90% coefficient interval (CI) to validate the test, defining a target efficacy for IVM as 99% against GINs in small ruminants extended to camelids [31,32]. Furthermore, EPG mean values were manually entered in the online software tool FECRT (https://www.fecrt.com/, version 1.1.0, accessed on 23 July 2025), and we calculated the anthelmintic efficacy for each ruminant farm that received IVM with appropriate EPG values to run FECRT [31,32]. Optimal IVM efficacy was defined as 99% FECRT, whereas a “grey zone” between 98 and 90% of FECRT was considered suboptimal efficacy but not marked resistance. FECRT values ≤ 89% were considered as indicative of resistance to IVM [31].

### 2.4. Molecular Identification of GIN Species

#### 2.4.1. DNA Extraction

Nematode eggs from fecal samples subjected to coprological analyses were recovered for DNA extraction. A modified version of the “Nemabiome” method [33] for nucleic acid extraction from nematode eggs was employed (https://www.nemabiome.ca/parasite, accessed on 23 July 2025). In short, approximately 200–1000 GIN eggs (with morula or larva inside) per sample were transferred to a 1.5 mL tube spin down, and 1.3 mL of a lysis buffer was added, mixed and incubated at room temperature for 5 min. After, the samples were centrifuged at 13,000× *g* for 4 min, discarding the supernatant and leaving the pellet, resuspension in a lysis buffer and pellet recovery for 3 times were repeated. Further, 50 μL of a lysis buffer was added for pellet resuspension. Later, the tube was incubated at 95 °C for 15 min, with vortexing every 3 min. Afterwards, the pellet was kept at −80 °C for 2 h and then thawed on ice. Next, 6 μL of proteinase K (20 mg/mL) was added to the tube and incubated at 55 °C for 2 h with regular vortexing every 1 min. Finally, the sample was incubated at 95 °C for 20 min to denature the proteinase K. The final DNA lysate was diluted at a ratio of 1:10 to use as a template for further experiments [33].

#### 2.4.2. PCR Amplification of GIN Species

Overlapping regions of the internal-transcribed-spacer genes 1 and 2 (ITS1 and ITS2, GenBank accession number: KY930444.1) were used as generic references for the identification of GIN species from the strongyloidea superfamily (https://lifemap.cnrs.fr/, accessed on 23 July 2025). Following the approach described by Bisset et al. 2014 [14], we carried out a nested PCR strategy, amplifying a first-round genetic amplicon of 370–398 bp that was used as a template with specific oligos to amplify target regions of the ITS-2 gene corresponding to the most common GIN species in grazing ruminants, such as *H. contortus*, *Teladorsagia circumcincta*, *Trichostrongylus axei*, *Trichostrongylus colubriformis*, *Trichostrongylus vitrinus*, *Chabertia ovina*, *Oesophagostomum venulosum*, *Cooperia curticei* and *Camelostrongylus mentulatus* [34,35]. The generic GIN amplicon was also used as a template to amplify specific regions of the ITS2 gene for *Marshallagia marshalli* (GenBank accession number: MT110920.1) [36]. To identify GIN species infecting domesticated camelids, we used the mitochondrial cytochrome c oxidase subunit 1 (COX-1) gene to find *Lamanema chavezi* (GenBank accession number: MG598421.1) in camelid farms [36]. PCR reagents (all from ThermoFischer Scientific, Burlington, ON, Canada) included MilliQ water, a 5× buffer PCR mix, 10 mM of dNTPs, 10 μM of Primers FW and RW, a DNA template comprising 1:10 dilutions and polymerase at 0.5 U/μL. Thermocycler (Bio-Rad T100^TM^, Saint-Laurent, QC, Canada) conditions were 95 °C for 3 min, followed by 40 cycles of the second denaturation at 98 °C for 30 s, annealing for 20 s and extension at 72 °C for 1 min, including a final extension of 10 min at 72 °C. PCR amplicons were analyzed on agarose gels and verified for their expected sizes. All primers and DNA fragment sizes for the amplification of generic or specific GIN sequences are listed in Table 2.

#### 2.4.3. Identification of *H. contortus* from Recovered Nematode Eggs

GIN eggs recovered from the fecal samples were subjected to both molecular identification of *H. contortus* (*HcITS2* amplification as described before) and fluorescent staining with lectin conjugated to peanut FITC (PN-FITC) [37]. Stained eggs were then observed by fluorescent microscopy at 470/480 excitation and 527/530 emission, confirming the presence of *H. contortus* by both methods. Fluorescence and phase-contrast images were analyzed with the Image J software version 1.54g: https://imagej.net/, accessed on 23 July 2025.

## 3. Results

### 3.1. Prevalence and Identification GIN Species in Ruminant Farms

Through microscopical and molecular analyses, we identified multiple GIN species in most of the grazing ruminant farms included in the study. Table 3 summarizes the subset of GIN species found on each farm through molecular analyses, while Table 4 presents the nematode eggs identified from the genera *Nematodirus* and *Trichuris* spp. The most prevalent GINs found across grazing ruminant farms were *H. contortus* and *T. vitrinus*, present in 5 out of 6 farms (83.3% prevalence). Next, *T. circumcincta*, *Nematodirus* spp. and *Trichuris* spp. (Table 4) were present in 4 out of 6 farms (66.6%), and *C. ovina* and *T. axei* were identified in 3 out of the 6 farms (50%). Lower frequencies were found for *O. venulosum* and *C. mentulatus* (2 out of 6 farms for 33.3%), while *T. colubriformis*, *C. curticei*, *L. chavezi* and *M. marshalli* were all detected in just one farm out of 6 (16.6%).

### 3.2. Microscopic Identification of H. contortus Eggs

**Figure 1:**
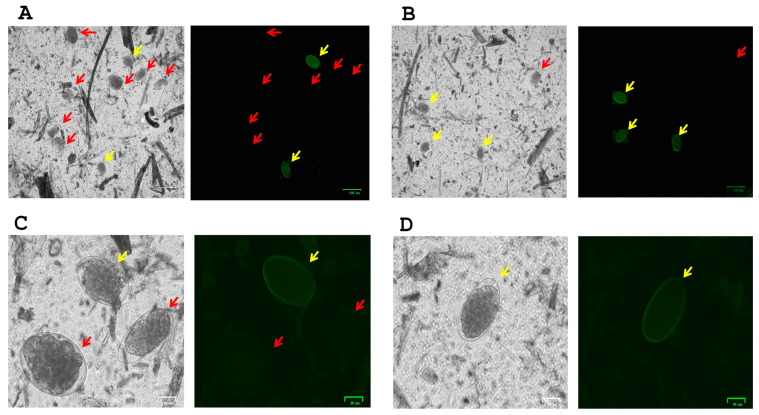
Fluorescence staining of *H. contortus* egg (yellow arrows) screening from other GIN spp. eggs (red arrows). Peanut agglutinin (PNA) fluorescent signal on *H. contortus* eggs was found in most farm samples, including sheep (**A**,**C**), sheep and llamas (**B**) and alpacas (**D**). Images (**A**,**B**) in bright field and FITC signal were captured at 10× magnification (scale bar at 100 μm), and images (**C**,**D**) in bright field and FITC signal were captured at 40× magnification (scale bar at 25 μm).

Using peanut agglutinin (PNA) conjugated to FITC for the staining of GIN eggs, we were able to detect the presence of *H. contortus* in 4 out 6 farms. Complementing our molecular identification of GIN species, we confirmed the presence of *H. contortus* among strongylid-like eggs in sheep farms (Figure 1A,C). Most of the samples showed a greater positive staining for the presence of *H. contortus* in farms with one or more ruminant species, including sheep and llamas from farm 1 (Figure 1B) and alpacas from farm 4 (Figure 1D).

### 3.3. FECs and IVM Efficacy on GINs from Grazing Ruminant Farms

The mean EPG with 90% CI was calculated for each sample collected before and after IVM treatment, as well as for untreated fecal samples. Strongylid-like eggs are described as “Total Strongylids”, while genera *Nematodirus* spp. and *Trichuis* spp. are reported separately. Table 5 presents the FEC values from sheep farm 1. In this farm, 11 animals older than 2 years old were included, and only 9 animals received IVM treatment, allowing for paired FECs at pre- and post-treatment comparisons. FEC values for GIN eggs showed a wide range of variation, fluctuating between 30 to more than 1000 EPG per animal at FEC only and pre-treatment samples. Egg counts for *Nematodirus* and *Trichuris* spp. maintained a low EGP, indicating a higher GIN burden of strongylid-like eggs, particularly from the *Trichostrongylidae* family.

Table 6 presents the FEC data from sheep farm 2. These values correspond to a pooled sample of 9 animals, aged 2 years and older, all of which were treated with IVM. Notably, the FECs revealed a high EPG mean at pre-treatment FEC for strongylid-like eggs, which was totally reduced (0 EPG) at post-treatment FEC. No eggs of *Nematodirus* or *Trichuris* spp. were detected.

Table 7 corresponds to the FECs from farm 3, including sheep and goats. Animals were not treated with IVM. Due to the small group sizes for both sheep and goats, we applied the Mini-FLOTAC method, which has a higher sensitivity to detect GIN infection in ruminants, compared with the FEC results from the Wisconsin method (see Supplementary Data S1). The FEC values indicate some moderate GIN burden, with each small ruminant group comprising more than 100 EGP means of strongylid-like eggs. Other nematode genera were scarcely detected by the Mini-FLOTAC method.

Table 8 summarizes the FECs from 2 llamas that were present in farm 1 that were also grazing with sheep. One llama (ID LAM 11) presented a moderate EPG for strongylid-like eggs at two FECs. The other llama (ID LAM12) showed an initial high EPG (pre-treatment) that was subjected to IVM treatment. However, comparing pre- and post-treatment samples for strongylid-like eggs, the difference in EPG means is reduced but not significant. For the other nematode genera as well, both animals show very low FECs.

FEC results from alpaca farm 4 that received a monthly treatment of IVM were divided by age. In Table 9, there are the individual FECs from the 1-year-old alpacas. EPG values fluctuated between pre- and post-treatment samples, independent of the method used (Mini-FLOTAC or Wisconsin; see Supplementary Data S2); however, due to the higher sensitivity to detecting *Nematodirus* and *Trichuris* eggs, we present the FECs from the Mini-FLOTAC method. Most animals exhibited low EPG values at post-treatment, which are recorded in the column labeled as “Total Strongylids”. Table 10 presents the FEC data for 2-year-old alpacas, which show non-significant EPG values overall, with only a few animals excreting more than 40 EPG during the pre-treatment assessment. Interestingly, in both age groups of alpacas, eggs of *Nematodirus* and *Trichuris* spp. were detected in 13 animals, with modest FECs (generally below 40 EPG). However, this represents a higher prevalence of these two nematode genera compared to the other farms included in our study.

We also included the FECs from pooled samples corresponding to two alpaca farms (farms 5 and 6, on Table 11) with different age subgroups. These two alpaca farms had no documented history of parasitism by GINs. After performing the coprology examinations, we found not significant FEC values for strongylid-like eggs nor for *Nematodirus* spp. and *Trichuris* spp. eggs. Based on this evidence, veterinarians and producers decided not to carry out an IVM treatment, opting instead to monitor using coprology analyses later in the season, beyond the timeframe of our study.

IVM efficacies were considered for only 3 out the 6 farms that received an IVM treatment and where the FEC values were suitable to calculate FECRT. Table 12 shows the efficacy results and classification from the FECRT analysis using the online software tool http://www.fecrt.com/, accessed on 23 July 2025. The results indicate that in farm 1 (sheep), there was a resistant status from GINs, as IVM treatment failed to significantly reduce the expected number of strongylid-like eggs. Both Delta and the classic WAAVP methods identified resistance through different statistical analyses. Nonetheless, the CI 90% upper limits of the tests are over 90%, placing the results in the “grey zone” or suboptimal efficacy, rather than clear resistance. Farm 2 (sheep) showed optimal IVM efficacy as the FECRT by the Delta and the BNB methods gave a clearly susceptible outcome for the GIN population subjected to IVM treatment. Although farm 2 corresponds to a pooled sample of sheep (N = 9, Table 6), the drastic reduction in EPG for the post-treatment sample was significant and validated by both Delta and BNB methods (Table 12).

In the case of both age groups from alpaca farm 4 (receiving monthly IVM treatment), notwithstanding the presence of an overall small EPG mean per animal, the FECRT analysis showed resistance for this farm, validated by the Delta and WAAVP methods, which showed that both CI 90% upper limits were below the threshold for the optimal efficacy window. We have included the full reports from the FECRT analyses run on the http://www.fecrt.com/, accessed on 23 July 2025. website as Supplementary Data (Data S3 for farm 1 (sheep); Data S4 are from farm 2 (sheep) and from alpaca farm 4 (monthly IVM treatment) for age groups of 1 year old (Data S5) and 2 years old (Data S6).

## 4. Discussion

Parasitic nematodes continue to hamper animal health in farm animals, such as grazing ruminants. Taxonomically and anatomically, ruminants such as cattle, sheep and goats are in a different Artiodactyla suborder from south American camelids (SACs, e.g., alpacas) based on the number of stomachs [40]. Nonetheless, from veterinary care and regulatory perspectives, SACs are included in the “ruminant” group, as they are raised in pastures for livestock production and share common infectious diseases, as illustrated by their susceptibility to endo-parasitoses for gastro-intestinal nematodes (GINs) [41]. Our study has examined the efficacy of ivermectin (IVM), the most common and available antiparasitic drug against GINs in livestock worldwide. In the ruminant farms included in our study, we identified several species of GINs affecting the animals and a varied efficacy of IVM to control these pathogens.

Our epidemiological data across most of the ruminant farms that included sheep, goats, llamas and alpacas reveal the presence of *H. contortus* and *T. vitrinus* as the most prominent GIN species infecting these ruminants. This is in line with the description of *H. contortus* as one of the most widespread endoparasites in ruminant livestock worldwide [42,43] including North America [23] Moreover, *H. contortus* has been extensively documented as one of the GINs that has developed anthelmintic resistance (AR) to all anthelmintic drug families, including the macrocyclic lactones (MLs) [44]. *T. vitrinus* has been described in small ruminants at different latitudes, such as Europe, the Middle East and Oceania [45,46,47], but there is no documented research on SACs such as that reported in our study. *T. vitrinus* has been described as a zoonotic helminth transmitted from ruminants to humans [48].

Besides *H. contortus* and *T. vitrinus*, our molecular analyses identified 9 other GIN species with strongylid-like eggs. In general, our results on the identification of GIN species from grazing ruminants have both similarities and contrasts with an earlier study specifically on sheep farms in Ontario and Québec [49]. Mederos et al. identified GIN species from larval cultures and morphological validation along with identification of adult worms from necropsies, whereas our study performed molecular analyses to establish GIN species in small ruminant and camelid farms. Although our study only comprised 6 ruminant farms, including 3 sheep farms with and without SACs, in both studies, *H. contortus* was found to be the most prevalent GIN species in sheep, whereas the other GIN species are described in the two studies at different prevalences.

Our assessment on the IVM efficacy against GINs in different ruminant farms showed a variable efficacy of this drug. The efficacy was determined through FECRT, including only suitable data from individual animals treated with IVM (Ivomec^®^) and counting with a minimal arithmetic mean of 40 EPG at pre-treatment FECs [31]. In the case of lower EPG means per animal, we considered a minimum of 5 animals per group, with an overall arithmetic mean of 15 EPG at pre-treatment FECs, as established by the WAAVP guidelines for anthelmintic efficacy field studies in ruminants [31]. Taking these parameters into consideration, we carried out an analysis of IVM efficacy through the FECRT online software (http://www.fecrt.com/, accessed on 23 July 2025) for only three farms treated with IVM. We found optimal IVM efficacy in only one sheep farm (farm 2 ≥ 99% FECRT, Table 12), whereas in another sheep farm (farm 1, Table 12), IVM efficacy was non-optimal and reported as IVM resistance; however, the CI 90% upper limits for two FECRT methods were between 90–99%, which is interpreted as being in the “grey zone” [31]. Results were validated through two statistical analyses (Table 12). This finding of loss of IVM efficacy in sheep farms has been well documented in North America and elsewhere [44,50,51].

Of interest, our study included an alpaca farm that administrates a monthly IVM treatment to camelids as a prophylactic against the meningeal worm *P. tenuis*. This practice is controversial in camelid farms in Eastern North America, including the US and Canada, as it may lead to selection of resistance of GINs to IVM and other MLs [52]. Our findings identified *H. contortus* and *T. vitrinus*, which have been under IVM selection, decreasing anthelmintic efficacy by up to a resistant status (Table 12, farm 4, both alpaca age groups). Resistance in *H. contortus* to MLs, including IVM, has been an emerging problem in the last 15 years in alpaca farms [53,54]. *Trichostrongylus* spp. resistance to IVM and to other anthelmintic classes has also been described in alpaca farms [55]. On the alpaca farm with monthly IVM treatment, we found limited IVM efficacy, indicating a selection process allowing for both *H. contortus* and *T. vitrinus* to develop an IVM-resistant phenotypes. Several studies have examined the basis of ML resistance in GINs, including *H. contortus* [16], but there is still no consensus; this is explained in part by the multigenic mechanism underlying AR to ML anthelmintics in veterinary nematodes [56]. The absence of molecular markers for resistance to MLs is another limitation in establishing a clear phenotype in ML-resistant parasitic helminths [16]. For these reasons, FECRT remains the most straightforward approach in field studies to assess anthelmintic efficacy against GINs in animal health, including farm ruminants raised in pastures.

## 5. Limitations of the Study

Although our study found the presence of IVM resistance in GINs from grazing ruminant farms in QC, Canada, using approved standardized analyses, we were careful about making assumptions about a general resistant process in the province’s livestock farms that are exposed to GINs, such *H. contortus*. Firstly, we only considered 6 ruminant farms, and further, only 3 have suitable data to assess IVM efficacy through FECRT. In addition, due to the overall medium–low EPG means per farm, we did not have enough parasite material to run the two coprological methods (Wisconsin and Mini-FLOTAC) and molecular analyses in all farms, such as farm 6 (alpacas), or to have parasite cultures to perform a bioassay to confirm IVM resistance in GINs through a larval developmental assay (LDA) or a larval migration assay (LMA) [57]. Another constraint of our study is that we applied the FECRT parameters from sheep to assess anthelmintic efficacy/AR in camelid farms. This is not ideal, but currently, there are no specific guidelines for studies of anthelmintic efficacy on SACs. Future studies should include a larger number of grazing ruminant farms and apply a consistent methodology (e.g., include only individual fecal samples vs pooled fecal samples) to validate anthelmintic efficacy studies by FECRT combined with pharmacological in vitro assays to clearly establish the presence of optimal drug efficacy or the presence of AR.

## 6. Conclusions

Our study has shown that grazing ruminants in Québec farms are exposed to several GINs, including *H. contortus*, a parasitic helminth species that may be becoming resistant to IVM. We found a variable efficacy of IVM against GINs, suggesting a process of parasite selection for resistance to IVM, and this phenomenon is independent of the ruminant species and age. All this information requires attention from veterinarians and livestock producers to direct applications of the best control strategies against these pathogens. Opting for the right choice of anthelmintic drug when deworming ruminants, coupled with alternative methods (refugia and pasture rotation, for instance), will help in the control of parasitic nematodes in grazing animal farms.

## Figures and Tables

**Table 1:** Detailed description of grazing ruminant farms included in the study.

Farm ID	Ruminant Species	Number of Animals and Age/Sex	Type of Fecal Sample Collected and Sampling Intervals	Number of Animals That Received IVM Treatment
1	Sheep and llamas (raised together)	11 sheep, 2 Y.O. (7 females and 4 males) 2 llamas, 2 Y.O. (2 males) N total: 11	Individual samples from 11 sheep (10 g each), pre- and 14 days post-IVM treatment. Individual samples from 4 llamas (20 g each), including 2 samples from 1 llama at pre- and post-IVM treatment.	9 sheep and 1 llama
2	Sheep	9 sheep, 2 Y.O. (9 females) N total: 9	Two pooled samples containing 10 g of feces from each animal. Each pooled sample corresponded to pre-and post-IVM treatment.	9 sheep
3	Goats and sheep (raised together)	5 goats, 2 Y.O. (5 females) 1 kid, 6 months old 2 sheep, 2 Y.O. (2 females) N total: 8	One pooled sample from goats, with 10 g from each animal. A total of 2–10 g of individual samples from each sheep.	No animal received treatment
4	Alpacas	2 groups: Group 1: 8 youngs, 1 Y.O. (4 females and 4 males) Group 2: 7 adults 2 Y.O. (6 females and 1 male) N total: 15	Individual samples (20 g) from each animal at pre- and 14 days post-IVM treatment. Pre-treatment samples were collected once at 30–31 days between IVM treatments.	15 alpacas
5	Alpacas	3 groups: Group 1: 6 female adults, 2 Y.O. Group 2: 3 male adults, 2 Y.O. Group 3: 1 young, 1 Y.O. Group 4: 1 cria, 6 months old N total: 11	Pooled samples from age groups 1 and 2, containing at least 5 g from each animal. Age groups 3 and 4 were collected individual samples (5 g).	No animal received treatment
6	Alpacas	3 groups: Group 1: 5 male adults, 2 Y.O. Group 2: 3 male adults, 2 Y.O. Group 3: 4 female adults, 2 Y.O. Group 4; 1 young, 1 Y.O. N total: 13	Pooled samples from each group, containing at least 5 g from each animal.	No animal received treatment

Footnote: Y.O.: year old.

**Table 2:** List of primers used for PCR amplification of genes from GIN species in ruminant farms.

GIN Species, Gene and Reference	Sequence (5′ → 3′)		PCR Product Size (bp)	Annealing Temp
GIN *generic ITS2* [35]	FW	CACGAATTGCAGACGCTTAG	370–398	53 °C
	RV	GCTAAATGATATGCTTAAGTTCAGC		
*H. contortus* (1) *ITS2* [35]	FW	CACGAATTGCAGACGCTTAG	170	53 °C
	RV	CTTGAACTGAAATGGGAATTGTCT		
*H. contortus ITS2* (2) [36]	FW	GTTACAATTTCATAACATCACGT	321	55 °C
	RV	TTTACAGTTTGCAGAACTTA		
*T. circumcincta ITS2* [36]	FW	ATACCGCATGGTGTGTACGG	421	58 °C
	RV	CAGGAACGTTACGACGGTAAT		
*T. axei ITS2* [36]	FW	AGGGATATTAATGTCGTTCA	67	56 °C
	RV	TGATAATTCCCATTTTAGTTT		
*T. colubriformis ITS2* [36]	FW	CCCGTTAGAGCTCTGTATA	165	59 °C
	RV	TGCGTACTCAACCACCACTAT		
*T. vitrinus ITS2* [36]	FW	AGGAACATTAATGTCGTTACA	100	54 °C
	RV	CTGTTTGTCGAATGGTTATTA		
*Ch. ovina ITS2* [36]	FW	CATGTGTGATCCTCGTACTAGATAAGA	158	54 °C
	RV	ATGAACCGTACACCGTTGTCA		
*O. venulosum ITS2* [36]	FW	TGTTTACTACAGTGTGGCTTG	280	54 °C
	RV	CGGTTGTCTCATTTCACAGGC		
*C. curticei ITS2* [36]	FW	TATACTACAGTGTGGCTAGCG	143	54 °C
	RV	TCATACCATTCAGAAATGTTC		
*C. mentulatus ITS2* (this study)	FW	CTTCGGCACGTCTGGTTCAG	278	55 °C
	RV	TGAGCTCAGGTTGCAATACAAA		
*M. marshalli COX1* (this study)	FW	TCATGAATGACACATGCAACA	188	53 °C
	RV	TAAGTTCAGCGGGTAATCACG		
*L. chavezi* *COX-1* (this study)	FW	TTTGGGCATCCTGAGGTTTA	157	53 °C
	RV	GAGCTCAAACCACACAACCA		

**Table 3:** GIN species identified by molecular analyses and their prevalences found in grazing ruminant farms.

GIN Species	Farm 1		Farm 2	Farm 3		Farm 4	Farm 5	Farm 6	Prevalence (%)
	Sheep N = 11	Llama N = 2	Sheep N = 9	Sheep N = 2	Goat N = 6	Alpaca N = 15	Alpaca N = 11	Alpaca N = 13	
*H. contortus*	pos	pos	pos	pos	pos	pos	pos	N/A	83.3%
*T. circumcincta*	pos	pos	pos	pos	neg	neg	neg	N/A	66.6%
*T. axei*	pos	pos	neg	pos	neg	neg	neg	N/A	50%
*T. colubriformis*	neg	neg	pos	neg	neg	neg	neg	N/A	16.6%
*T. vitrinus*	pos	pos	pos	pos	pos	pos	pos	N/A	83.3%
*O. venulosum*	pos	neg	neg	neg	neg	neg	pos	N/A	33.3%
*C. curticei*	pos	neg	neg	neg	neg	neg	neg	N/A	16.6%
*C. ovina*	pos	pos	neg	pos	neg	neg	neg	N/A	50%
*C. mentulatus*	N/A	neg	N/A	N/A	N/A	pos	pos	N/A	33.3%
*L. chavezi*	N/A	neg	N/A	N/A	N/A	pos	neg	N/A	16.6%
*M. marshalli*	N/A	neg	N/A	N/A	N/A	pos	neg	N/A	16.6%

Footnote: pos or neg for positive or negative identification. N/A: not applicable.

**Table 4:** GIN genera *Nematodirus* spp. and *Trichuris* spp., identified by microscopic analysis in grazing ruminant farms.

Genera	Farm 1		Farm 2	Farm 3		Farm 4	Farm 5	Farm 6	Prevalence (%)
	Sheep N = 11	Llama N = 2	Sheep N = 9	Sheep N = 2	Goat N = 6	Alpaca N = 15	Alpaca N = 11	Alpaca N = 13	
*Nematodirus* spp.	pos	pos	neg	neg	neg	pos	pos	pos	66.6%
*Trichuris* spp.	pos	pos	neg	pos	neg	pos	pos	neg	66.6%

Footnote: pos or neg for positive or negative identification. Coprological analysis by Wisconsin method on farms 1, 2, 5 and 6 and by Wisconsin and Mini-FLOTAC methods on farms 3 and 4.

**Table 5:** Individual FECs from sheep farm 1. Animals were 2 years and older.

Farm	Sample ID	Type	Total Strongylids		*Nematodirus* spp.		*Trichuris* spp.	
			EPG Mean	SD (90% CI)	EPG Mean	SD (90% CI)	EPG Mean	SD (90% CI)
1	SHP 11	FEC	138.6	±22.2 (102.0–175.1)	3.7	±2.1	0	N/A
1	SHP 12	FEC	22.3	±4.4 (15.0–29.5)	0.7	±1.2	0	N/A
1	SHP 13	pre-treatment	61.7	±34.5 (4.9–118.4)	0.3	±0.3 (−0.1–0.7)	0.1	±0.2
		post-treatment	12.2	±3.6 (6.1–18.2)	0	N/A	0	N/A
1	SHP 14	pre-treatment	120.4	±28.2 (74.0–166.7)	2.3	±0.3 (1.8–2.7)	1.6	±0.7 (0.4–2.7)
		post-treatment	5.4	±1.4 (3.0–7.7)	0	N/A	0	N/A
1	SHP 15	pre-treatment	31.9	±14.8 (7.5–56.2)	0	N/A	0	N/A
		post-treatment	0.6	±0.2 (0.2–0.9)	0	N/A	0	N/A
1	SHP 16	pre-treatment	35.2	±22 (−0.9–71.3)	0	N/A	0	N/A
		post-treatment	16.3	±5.6 (7.0–25.5)	0.3	±0.34 (−0.2–1.8)	0	NA
1	SHP 17	pre-treatment	1013.2	±139.7 (783.4–1242.9)	0	NA	0	N/A
		post-treatment	31.9	±10 (14.3–49.5)	0	N/A	0	N/A
1	SHP 18	pre-treatment	248	±33.4 (193.0–302.9)	0.3	±0.34 (−0.2–1.8)	0.4	±0.57 (−0.5–1.3)
		post-treatment	1.8	±1.1 (−0.0–3.6)	0	N/A	0	N/A
1	SHP 19	pre-treatment	239.4	±239 (−153.7–632.5)	0	N/A	0	N/A
		post-treatment	0.1	±0.2 (−0.2–0.4)	0	N/A	0	N/A
1	SHP 20	pre-treatment	594.1	±175.8 (304.9–883.2)	3.1	±0.4 (2.4–3.7)	0.6	±0.2 (0.2–0.9)
		post-treatment	337	±110.9 (171.0–502.9)	7.1	±2.5 (2.9–11.2)	0.3	±0.3 (−0.1–0.7)
1	SHP 21	pre-treatment	1019.1	±268.3 (577.7–1460.4)	61.1	±13.6 (38.7–83.4)	1.8	±0.4 (1.1–2.4)
		post-treatment	335.8	±355.1 (−248.2–919.8)	0.8	±1.1 (−1.0–2.6)	0	N/A

Footnote: EPG: eggs per gram. FEC: fecal egg count. SD: standard deviation. 90% CI (coefficient interval). N/A: non-applicable. Coprological analysis by Wisconsin method.

**Table 6:** Pooled FECs from sheep farm 2. Animals were 2 years and older.

Farm	Sample ID	Type	Total Strongylids		*Nematodirus* spp.		*Trichuris* spp.	
			EPG Mean	SD (90% CI)	EPG Mean	SD (90% CI)	EPG Mean	SD (90% CI)
2	SHP 30a Adults N = 9	pre-treatment	714.2	±142.4 (479.9–948.4)	0	N/A	0	N/A
		post-treatment	0	NA	0	N/A	0	N/A

Footnote: EPG: eggs per gram. SD: standard deviation. 90% CI (coefficient interval). N/A: non-applicable. Coprological analysis by Wisconsin method.

**Table 7:** Pooled FECs from goat and individual FECs from sheep farm 3.

Farm	Sample ID	Type	Total Strongylids		*Nematodirus* spp.		*Trichuris* spp.	
			EPG Mean	SD (90% CI)	EPG Mean	SD (90% CI)	EPG Mean	SD (90% CI)
3	GOT 11 (5 females and 1 kid) N = 6	FEC	416	±35.1 (393.1–440.2)	0.0	N/A	0.0	N/A
3	Sheep 11 (1 adult, 2-years old)	FEC	113.3	±55.1 (22.6–203.9)	0	N/A	0	N/A
3	Sheep 12 (1 adult, 2-years old)	FEC	196.7	±47.3 (118.8–274.5)	0.8	±1.24 (−1.2–2.8)	0	N/A

Footnote: EPG: eggs per gram. FEC: fecal egg count. SD: standard deviation. 90% CI (coefficient interval). N/A: non-applicable. Coprological analysis by Mini-FLOTAC method.

**Table 8:** Individual FECs from llamas from farm 1. Animals were 2 years old.

Farm	Sample ID	Type	Total Strongylids		*Nematodirus* spp.		*Trichuris* spp.	
			EPG Mean	SD (90% CI)	EPG Mean	SD (90% CI)	EPG Mean	SD (90% CI)
1	LAM 11	FEC	76.4	±27.0 (31.8–120.8)	2.0	±1.0 (0.3–3.6)	0.0	N/A
		FEC	21.3	±11.9 (1.7–40.8)	1.8	±1.2 (−0.1–3.7)	0.1	±0.2 (−0.2–0.4)
1	LAM 12	pre-treatment	526.6	±347.4 (−44.8–1098.0)	1.0	±1.2 (−0.9–2.9)	0.1	±0.2 (−0.2–0.4)
		post-treatment	185.2	±109.8 (4.5–365.8)	0.0	N/A	0.0	N/A

Footnote: EPG: eggs per gram. FEC: fecal egg count. SD: standard deviation. 90% CI (coefficient interval). N/A: non-applicable. Coprological analysis by Wisconsin method.

**Table 9:** Individual FECs from farm 4 corresponding to 1-year-old alpacas that received a monthly treatment with IVM.

Farm	Sample ID	Type	Total Strongylids		*Nematodirus* spp.		*Trichuris* spp.	
			EPG Mean	SD (90% CI)	EPG Mean	SD (90% CI)	EPG Mean	SD (90% CI)
4	ALP 11	pre-treatment	96.7	±67 (−13.2–207.2)	6.7	±5.7 (−2.6–16.0)	7	±5.9 (−2.7–16.7)
		post-treatment	153.3	±50 (71.0–235.5)	20	±10 (3.5–36.4)	10	±10 (−6.4–26.4)
4	ALP 12	pre-treatment	30	±17 (2.0–57.9)	0	N/A	20	±17.3 (−15.5–50.1)
		post-treatment	23.3	±12.1 (3.3–43.2)	0	N/A	10	±10 (−6.4–26.4)
4	ALP 13	pre-treatment	3.3	±6.2 (−7.1–13.1)	0	N/A	0	N/A
		post-treatment	0	N/A	0	N/A	0	N/A
4	ALP 14	pre-treatment	146.7	±49.4 (65.4–227.9)	3.3	±5.7 (−6.0–12.6)	23.3	±10 (−6.4–26.4)
		post-treatment	6.7	±5.7 (−2.6–16.0)	0	N/A	3.3	±5.7 (−6.0–12.6)
4	ALP 15	pre-treatment	16.7	±5.7 (7.3–26.0)	0	N/A	0	N/A
		post-treatment	16.7	±11.5 (−2.2–35.6)	0	N/A	3.3	±5.7 (−6.0–12.6)
4	ALP 16	pre-treatment	23.3	±32 (−29.3–75.9)	20	±34.6 (−36.9–76.9)	0	NA
		post-treatment	13.3	±5.7 (3.9–22.6)	10	N/A	3.3	±5.7 (−6.0–12.6)
4	ALP 17	pre-treatment	45	±35.3 (−13.0–103.0)	10	±14.1 (−13.1–33.1)	10	NA
		post-treatment	53.3	±32.1 (0.5–106.1)	40	±30.5 (−10.1–90.1)	10	±10 (−6.4–26.4)
4	ALP 18	pre-treatment	6.7	±5.7 (−2.6–16.0)	3.3	±5.7 (−6.0–12.6)	3.3	±5.7 (−6.0–12.6)
		post-treatment	26.7	±15.2 (1.6–51.7)	23.3	±11.5 (4.3–42.2)	3.3	±5.7 (−6.0–12.6)

Footnote: EPG: eggs per gram. SD: standard deviation. 90% CI (coefficient interval). N/A: non-applicable. Coprological analysis by Mini-FLOTAC method.

**Table 10:** Individual FECs from farm 4 corresponding to 2-year-old alpacas that received a monthly treatment with IVM.

Farm	Sample ID	Type	Total Strongylids		*Nematodirus* spp.		*Trichuris* spp.	
			EPG Mean	SD (90% CI)	EPG Mean	SD (90% CI)	EPG Mean	SD (90% CI)
4	ALP 20	pre-treatment	0	N/A	0	N/A	0	N/A
		post-treatment	6.7	±5.7 (−2.6–16.0)	0	N/A	0	N/A
4	ALP 21	pre-treatment	10.2	NA	0	NA	0	N/A
		post-treatment	27.1	±11.5 (12.2–25.6)	3.3	±5.7 (−6.0–12.6)	0	N/A
4	ALP 22	pre-treatment	32.8	NA	0	N/A	0	N/A
		post-treatment	56.7	NA	0	N/A	0	N/A
4	ALP 23	pre-treatment	43.3	±25.1 (2.0–84.5)	0	N/A	0	N/A
		post-treatment	23.3	NA	0	N/A	0	N/A
4	ALP 24	pre-treatment	10	±10 (−6.4–26.4)	0	N/A	10	±10 (−6.4–26.4)
		post-treatment	3.3	±5.7 (−6.0–12.6)	0	N/A	3.3	±5.7 (−6.0–12.6)
4	ALP 25	pre-treatment	43.3	±15.2 (18.2–68.3)	36.7	±11.5 (17.7–55.6)	0	N/A
		post-treatment	20	NA	10	±10 (−6.4–26.4)	0	N/A
4	ALP 26	pre-treatment	23.3	±20.8 (−10.9–57.5)	0	N/A	16.7	±15.2 (−8.3–41.7)
		post-treatment	10	±17.3 (−18.4–38.4)	0	N/A	3.3	±5.7 (−6.0–12.6)

Footnote: EPG: eggs per gram. SD: standard deviation. 90% CI (coefficient interval). NA: non-applicable. Coprological analysis by Mini-FLOTAC method.

**Table 11:** Pooled FECs from alpaca farms 5 and 6.

Farm	Sample ID	Type	Total Strongylids		*Nematodirus* spp.		*Trichuris* spp.	
			EPG Mean	SD (90% CI)	EPG Mean	SD (90% CI)	EPG Mean	SD (90% CI)
5	ALP (females, 2 Y.O.) N = 6	FEC	35.7	±6.3 (25.3–46.0)	2.2	±1.2 (0.2–4.1)	0.2	±0.1 (0.0–0.3)
5	ALP (old males, 2 Y.O.) N = 3	FEC	2.8	±1.1 (0.9–4.6)	0.6	±0.6 (−0.3–1.5)	0.1	±0.1 (−0.0–0.2)
5	ALP (young male, 1 Y.O.) N = 1	FEC	1.3	±0.5 (0.4–2.1)	0.7	±0.4 (0.0–1.3)	0.1	±0.1 (−0.0–0.2)
5	ALP (crias, <1 Y.O.) N = 1	FEC	1	±1.1 (−0.8–2.8)	0.6	±0.6 (−0.3–1.5)	0.2	±0.1 (−0.0–0.3)
6	ALP (young, 1 Y.O.) N = 5	FEC	8	±1.0 (6.3–9.6)	0	NA	0.3	±0.3 (−0.1–0.7)
6	ALP males (1) (2 Y.O) N = 5	FEC	11.9	±6.8 (0.7–23.0)	0.1	±0.1 (−0.0–0.2)	0.1	±0.1 (−0.0–0.2)
6	ALP males (2) (2 Y.O) N = 3	FEC	1.6	±1.5 (−0.8–4.0)	0	NA	1.3	±1.3 (−0.8–3.4)
6	ALP (females, 2 Y.O.) N = 4	FEC	4.1	±1.7 (1.3–6.8)	0.1	±0.1 (−0.1–0.3)	3	±1.1 (1.1–4.8)

Footnote: EPG: eggs per gram. FEC: fecal egg count. SD: standard deviation. 90% CI (coefficient interval). NA: non-applicable. Y.O.: year-old. Coprological analysis by Wisconsin method.

**Table 12:** Efficacy of IVM against GINs (strongylid-like eggs only) based on FECRT *.

Efficacy Classification	Expected Efficacy for IVM (MLs) **	Delta Method *** (Levecke et al., 2018) [38]	WAAVP Method *** (Coles et al., 2006) [39]	BNB Method *** (Denwood et al., 2023) [31]
Farm 1 (adult sheep) N = 9	99% with lower threshold of 90%	Resistant 90% CI = 48.8–95.9	Resistant 90% CI = 21–93.9	Unavailable
Farm 2 (adult sheep) N = 9	99% with lower threshold of 90%	Susceptible 90% CI = NA	Unavailable	Susceptible Version B ^#^: *p* < 0.001
Farm 4 (alpacas, 1 Y.O.) N = 8	99% with lower threshold of 90%	Resistant 90% CI = −82.1–79.8	Resistant 90% CI = −162.7–73.4	Unavailable
Farm 4 (alpacas, 2 Y.O.) N = 6	99% with lower threshold of 90%	Resistant 90% CI = −49.7–61.4	Resistant 90% CI = −92.9–61.3	Unavailable

Footnotes: * IVM efficacy analysis was run on the online software http://www.fecrt.com/, accessed on 23 July 2025, including pairing samples (pre- and post-IVM treatment). 90% CI (coefficient interval). ** Expected IVM efficacy in sheep [32] and extended to south American camelids (alpacas). *** FECRT methods applied according to the WAAVP guidelines, Kaplan et al., 2023 [32]. ^#^ FECRT method applied to group of animals when N ≥ 5.

## Data Availability

All data included from this research is enclosed in this research article.

## Supplementary Materials

The following supporting information can be downloaded at: https://www.mdpi.com/article/10.3390/pathogens14100984/s1, Data S1. FEC data from farm 3 (goats and sheep) using Wisconsin method (pdf). Data S2. FEC data from farm 4 (alpacas), both group ages (1 Y.O) and 2 Y.O, at both pre- and post-IVM treatment. (pdf). Data S3. Full FECRT report on farm 1 (sheep) from the online software http://fecrt.com/ (pdf). Data S4. Full FECRT report on farm 2 (sheep) from the online software http://fecrt.com/ (pdf). Data S5. Full FECRT report on farm 4 (alpaca, 1 Y.O. group) from the online software http://fecrt.com/ (pdf). Data S6. Full FECRT report on farm 4 (alpaca, 2 Y.O. group) from the online software http://fecrt.com/ (pdf).
