# Supplementary material for: Coprological and Molecular Analyses of Ruminant Farms in Québec, Canada, Show a Variable Efficacy of Ivermectin Against Gastro-Intestinal Nematodes
Source: Pathogens. 2025 Sep 28;14(10):984. doi: 10.3390/pathogens14100984 (PMC12567335; doi:10.3390/pathogens14100984)
Supplement: Supplementary file 1 [file pathogens-14-00984-s001.zip › Data S1. FEC data from farm 3 (goats and sheep) using Wis-consin method.pdf]

Supplementary data S1. Pool FECs from goats and invidual FEC from sheep farm 3. Wisconsin method

Animal ID			GIN eggs	EPG	EPG Mean	SD	*Trichuris*	EPG	EPG Mean	CI 95%	*Nematodirus*	EPG	EPG Mean	CI 95%
Sheep 11b (1 adult 2 years-old)	Replicate 1	Chamber 1	2	50	66.7	20.8	0	0	0.0	0.0	0	0	0	0.0
		Chamber 2	3				0				0			
	Replicate 2	Chamber 1	6	90			0	0			0	0		
		Chamber 2	3				0				0			
	Replicate 3	Chamber 1	2	60			0	0			0	0		
		Chamber 2	4				0				0			
Got 11a (5 females and 1 kid N=6)	Replicate 1	Chamber 1	6	110	163.3	83.9	0	0	0	0.0	0	0	0	0.0
		Chamber 2	5				0				0			
	Replicate 2	Chamber 1	5	120			0	0			0	0		
		Chamber 2	7				0				0			
	Replicate 3	Chamber 1	9	260			0	0			0	0		
		Chamber 2	17				0				0			
Sheep 11c (1 adult 2 years-old)	Replicate 1	Chamber 1	1	30	73.3	51.3	0	0	0	0.0	0	0	0	0.0
		Chamber 2	2				0				0			
	Replicate 2	Chamber 1	2	60			0	0			0	0		
		Chamber 2	4				0				0			
	Replicate 3	Chamber 1	5	130			0	0			0	0		
		Chamber 2	8				0				0			
