# Supplementary material for: Coprological and Molecular Analyses of Ruminant Farms in Québec, Canada, Show a Variable Efficacy of Ivermectin Against Gastro-Intestinal Nematodes
Source: Pathogens. 2025 Sep 28;14(10):984. doi: 10.3390/pathogens14100984 (PMC12567335; doi:10.3390/pathogens14100984)
Supplement: Supplementary file 1 [file pathogens-14-00984-s001.zip › Data S2. FEC data from farm 4 (alpacas), both group ages (1 Y.O) and 2 Y.O, at both pre- and post-IVM treatment.pdf]

Supplementary data S2. Alpaca farm 4 fecal egg count report. Wisconsin mtethod. Alpaca FEC report 1.Y.O. Pre-treatment

Animal ID			Strongylid eggs	EPG	EPG Mean	SD	CI 90%	Nematodirus	EPG	EPG Mean	SD	CI 90%	Trichuris	EPG	EPG Mean	SD	CI 90%
**ALP11**	Replicate 1	Chamber 1	1	80	**46.7**	± 30.5	± 28.9	1	10	**3.3**	**±5.7**	**± 5.4**	0	0	**0.0**	**±0.0**	**± 0**
		Chamber 2	7					0					0				
	Replicate 2	Chamber 1	0	20				0	0				0	0			
		Chamber 2	2					0					0				
	Replicate 3	Chamber 1	2	40				0	0				0	0			
		Chamber 2	2					0					0				
**ALP12**	Replicate 1	Chamber 1	0	10	**6.7**	± 5.7	± 5.4	0	0	**0.0**	**±0.0**	**± 0**	0	0	**0.0**	**±0.0**	**± 0**
		Chamber 2	1					0					0				
	Replicate 2	Chamber 1	1	10				0	0				0	0			
		Chamber 2	0					0					0				
	Replicate 3	Chamber 1	0	0				0	0				0	0			
		Chamber 2	0					0					0				
**ALP13**	Replicate 1	Chamber 1	0	0	**6.7**	± 5.7	± 5.4	0	0	**0.0**	**±0.0**	**± 0**	0	0	**0.0**	**±0.0**	**± 0**
		Chamber 2	0					0					0				
	Replicate 2	Chamber 1	1	20				0	0				0	0			
		Chamber 2	1					0					0				
	Replicate 3	Chamber 1	0	0				0	0				0	0			
		Chamber 2	0					0					0				
**ALP14**	Replicate 1	Chamber 1	6	120	**60.0**	± 52.9	±50.2	0	0	**0.0**	**±0.0**	**± 0**	0	0	**0.0**	**±0.0**	**± 0**
		Chamber 2	6					0					0				
	Replicate 2	Chamber 1	3	40				0	0				0	0			
		Chamber 2	1					0					0				
	Replicate 3	Chamber 1	0	20				0	0				0	0			
		Chamber 2	2					0					0				
**ALP15**	Replicate 1	Chamber 1	0	10	**6.7**	± 5.7	± 5.4	0	0	**0.0**	**±0.0**	**± 0**	0	0	**0.0**	**±0.0**	**± 0**
		Chamber 2	1					0					0				
	Replicate 2	Chamber 1	1	10				0	0				0	0			
		Chamber 2	0					0					0				
	Replicate 3	Chamber 1	0	0				0	0				0	0			
		Chamber 2	0					0					0				
**ALP16**	Replicate 1	Chamber 1	0	10	**6.7**	± 5.7	± 5.4	1	20	**16.7**	**±5.7**	**± 5.4**	0	0	**0.0**	**±0.0**	**± 0**
		Chamber 2	1					1					0				
	Replicate 2	Chamber 1	0	0				1	10				0	0			
		Chamber 2	0					0					0				
	Replicate 3	Chamber 1	1	10				1	20				0	0			
		Chamber 2	0					1					0				
**ALP17**	Replicate 1	Chamber 1	2	20	**16.7**	**± 15.2**	± 14.4	0	20	**23.3**	**±5.7**	**± 5.4**	0	0	**0.0**	**±0.0**	**± 0**
		Chamber 2	0					2					0				
	Replicate 2	Chamber 1	1	30				1	30				0	0			
		Chamber 2	2					2					0				
	Replicate 3	Chamber 1	0	0				0	20				0	0			
		Chamber 2	0					2					0				
	Replicate 1	Chamber 1	0	10				0	10				0	0			

**ALP18**	Replicate 1	Chamber 2	1	10	**13.3**	**± 5.7**	**± 5.4**	1	10	**6.7**	**±5.7**	**± 5.4**	0	0	**0.0**	**±0.0**	**± 0**
	Replicate 2	Chamber 1	2	20				1	10				0	0			
		Chamber 2	0					0					0				
	Replicate 3	Chamber 1	0	10				0	0				0	0			
		Chamber 2	1					0					0				
