# Supplementary material for: Coprological and Molecular Analyses of Ruminant Farms in Québec, Canada, Show a Variable Efficacy of Ivermectin Against Gastro-Intestinal Nematodes
Source: Pathogens. 2025 Sep 28;14(10):984. doi: 10.3390/pathogens14100984 (PMC12567335; doi:10.3390/pathogens14100984)
Supplement: Supplementary file 1 [file pathogens-14-00984-s001.zip › Data S4. Full FECRT report on farm 2 (sheep) from online software fecrt.pdf]

Analysis of FECRT data from

fecrt.com data analysis tool

2025-07-10

Efficacy classification

Efficacy classification: Susceptible

[Based on an expected efficacy of 99% and a lower efficacy threshold of 90%]

WARNING: your data has fewer than five observations, so the classification above should be interpreted with extreme care!

Detailed results

The statistical method automatically chosen for your dataset was the BNB method (version C): see further details of statistical methods below the summary statistics

Summary statistics

Number of animals: 1

Mean of pre-treatment data: 714

Mean of post-treatment data: 0

Variance of pre-treatment data: (undefined)

Variance of post-treatment data: (undefined)

Estimated over-dispersion (k) of pre-treatment data: (undefined)

Estimated over-dispersion (k) of post-treatment data: (undefined)

Estimated within-animal correlation: (undefined)

Results from the Delta method (Levecke et al.)

Classification: Method_Failure

[90% CI uncalculable]

Notes:

- This method is non-parametric, so is robust to distributional assumptions, and variances of the pre-treatment and post-treatment data are estimated independently
- This method cannot be used when the post-treatment data are all zero
- This method may give misleading results with fewer than five observations, and when fewer than three post-treatment observations are non-zero, due to unstable variance estimates
- This is the preferred method when the sample size is greater than or equal to 5, and where at least three post-treatment observations are non-zero

Results from the WAAVP method (Coles et al. and Pepper et al.)

Classification: Method_Failure

[90% CI uncalculable]

Notes:

- This method is non-parametric, so is robust to distributional assumptions, and variances of the pre-treatment and post-treatment data are estimated independently
- This method cannot be used when the post-treatment data are all zero
- This method may give misleading results with fewer than five observations, and when fewer than three post-treatment observations are non-zero, due to unstable variance estimates

Results from the BNB method (Denwood et al.) version A

Classification: Susceptible

Test for Resistance: $p = 1.000$; Test for Susceptibility: $p < 0.001$

Notes:

- This method is parametric, and assumes that the data follow a negative binomial distribution: the classification will be unavailable if the multiplication factor you entered does not match the data
- The over-dispersion is estimated independently for the pre-treatment and post-treatmentdata
- This method may give misleading results with fewer than five observations, and when fewer than three post-treatment observations are non-zero, due to unstable estimates of over-dispersion

Results from the BNB method (Denwood et al.) version B

Classification: Susceptible

Test for Resistance: $p = 1.000$; Test for Susceptibility: $p < 0.001$

Notes:

- This method is parametric, and assumes that the data follow a negative binomial distribution: the classification will be unavailable if the multiplication factor you entered does not match the data
- The over-dispersion is estimated for the pre-treatment data, but the over-dispersion in the post-treatmentdata is assumed to be proportional to that of the pre-treatmentdata (the ratio used is based on published estimates of over-dispersion ratios in the host/parasite species you have selected)
- This method may give misleading results with fewer than five observations due to unstable estimates of over-dispersion
- This is the preferred method when the sample size is greater than or equal to 5, and where fewer than three post-treatment observations are non-zero

Results from the BNB method (Denwood et al.) version C

Classification: Susceptible

Test for Resistance: $p = 1.000$; Test for Susceptibility: $p = 0.013$

Notes:

- This method is parametric, and assumes that the data follow a negative binomial distribution: the classification will be unavailable if the multiplication factor you entered does not match the data
- The over-dispersion is not estimated from the data, but is assumed to follow published estimates for typical over-dispersion in the host/parasite species you have selected
- This method may give misleading results in some groups of animals where the population over-dispersion is in fact different to published estimates

- This is the only preferred method when the sample size is less than 5 (and is the only viable method with a sample size of 1)
