# Supplementary material for: Coprological and Molecular Analyses of Ruminant Farms in Québec, Canada, Show a Variable Efficacy of Ivermectin Against Gastro-Intestinal Nematodes
Source: Pathogens. 2025 Sep 28;14(10):984. doi: 10.3390/pathogens14100984 (PMC12567335; doi:10.3390/pathogens14100984)
Supplement: Supplementary file 1 [file pathogens-14-00984-s001.zip › Data S6. Full FECRT report on farm 4 (alpaca, 2 Y.O. group) from online software fecrt.com.pdf]

Analysis of FECRT data from

fecrt.com data analysis tool

2025-09-04

Efficacy classification

Efficacy classification: Resistant

[Based on an expected efficacy of 99% and a lower efficacy threshold of 90%]

Detailed results

The statistical method automatically chosen for your dataset was the Delta method: see further details of statistical methods below the summary statistics

Summary statistics

Number of animals: 6

Mean of pre-treatment data: 27

Mean of post-treatment data: 23.33

Variance of pre-treatment data: 228.4

Variance of post-treatment data: 349.87

Estimated over-dispersion (k) of pre-treatment data: 4.6

Estimated over-dispersion (k) of post-treatment data: 2.17

Estimated within-animal correlation: 0.33

Results from the Delta method (Levecke et al.)

Classification: Resistant

90% CI = -49.7% - 61.4%

Notes:

- This method is non-parametric, so is robust to distributional assumptions, and variances of the pre-treatment and post-treatment data are estimated independently
- This method cannot be used when the post-treatment data are all zero
- This method may give misleading results with fewer than five observations, and when fewer than three post-treatment observations are non-zero, due to unstable variance estimates
- This is the preferred method when the sample size is greater than or equal to 5, and where at least three post-treatment observations are non-zero

Results from the WAAVP method (Coles et al. and Pepper et al.)

Classification: Resistant

90% CI = -92.9% - 61.3%

Notes:

- This method is non-parametric, so is robust to distributional assumptions, and variances of the pre-treatment and post-treatment data are estimated independently
- This method cannot be used when the post-treatment data are all zero
- This method may give misleading results with fewer than five observations, and when fewer than three post-treatment observations are non-zero, due to unstable variance estimates

Results from the BNB method (Denwood et al.) version A

Classification: Unavailable (non-integer counts detected)

[test p-values uncalculable]

Notes:

- This method is parametric, and assumes that the data follow a negative binomial distribution: the classification will be unavailable if the multiplication factor you entered does not match the data
- The over-dispersion is estimated independently for the pre-treatment and post-treatmentdata
- This method may give misleading results with fewer than five observations, and when fewer than three post-treatment observations are non-zero, due to unstable estimates of over-dispersion

Results from the BNB method (Denwood et al.) version B

Classification: Unavailable (non-integer counts detected)

[test p-values uncalculable]

Notes:

- This method is parametric, and assumes that the data follow a negative binomial distribution: the classification will be unavailable if the multiplication factor you entered does not match the data
- The over-dispersion is estimated for the pre-treatment data, but the over-dispersion in the post-treatmentdata is assumed to be proportional to that of the pre-treatmentdata (the ratio used is based on published estimates of over-dispersion ratios in the host/parasite species you have selected)
- This method may give misleading results with fewer than five observations due to unstable estimates of over-dispersion
- This is the preferred method when the sample size is greater than or equal to 5, and where fewer than three post-treatment observations are non-zero

Results from the BNB method (Denwood et al.) version C

Classification: Unavailable (non-integer counts detected)

[test p-values uncalculable]

Notes:

- This method is parametric, and assumes that the data follow a negative binomial distribution: the classification will be unavailable if the multiplication factor you entered does not match the data
- The over-dispersion is not estimated from the data, but is assumed to follow published estimates for typical over-dispersion in the host/parasite species you have selected
- This method may give misleading results in some groups of animals where the population over-dispersion is in fact different to published estimates

- This is the only preferred method when the sample size is less than 5 (and is the only viable method with a sample size of 1)
